# A new species of *Paramunida* Baba, 1988 from the Central Pacific Ocean and a new genus to accommodate *P. granulata* (Henderson, 1885)

**DOI:** 10.3897/zookeys.425.7882

**Published:** 2014-07-10

**Authors:** Patricia Cabezas, Enrique Macpherson

**Affiliations:** 1 Smithsonian Institution, National Museum of Natural History, Department of Invertebrate Zoology, Museum Support Center, MRC 534, 4210 Silver Hill Road. Suitland, MD 20746; 2 Centre d’Estudis Avançats de Blanes (CEAB-CSIC), C. d’Accés Cala Sant Francesc 14, 17300 Blanes, Spain

**Keywords:** New species, *Paramunida*, new genus, *Hendersonida*, *Munididae*, squat lobster, morphology, phylogeny

## Abstract

The genus *Paramunida* belongs to the most diverse family of galatheoids and it is commonly reported from the continental slope across the Indian and Pacific Oceans. Examination of material collected by the NOAA RV *Townsend Cromwell* Cruise near Christmas (Kiritimati) Island, Kiribati, revealed the existence of a new species of *Paramunida* (*P. haigae*), which represents the fourth record of the genus for the Central Pacific. Furthermore, recent efforts to unravel phylogenetic relationships and diversification patterns in *Paramunida* revealed *P. granulata* (Henderson, 1885) to be the most basally diverging taxon within the genus. This species is clearly distinguished from other species of *Paramunida* by the spinulation of the carapace and the length of the distomesial spine of the second antennal peduncle article, which in combination with a high level of genetic divergence suggest that this species represents a separate monotypic lineage. A new genus, *Hendersonida* **gen. n.**, is proposed to accommodate this species based on morphological and molecular evidence. An updated dichotomous identification key for all species of *Paramunida* is presented.

## Introduction

Squat lobsters are abundant and highly visible crustaceans in the deep sea (Baba et al. 2008). Our understanding of the taxonomy and phylogeny of this speciose group has been revolutionized in the last three decades, mainly thanks to the numerous MUSORSTOM-TDSB expeditions (Richer de Forges et al. 2013). Major rearrangements at higher classifications (Ahyong et al. 2010; Schnabel and Ahyong 2010), the description of 14 new genera (Macpherson and Baba 2011; Macpherson and Robainas-Barcia 2013) and many new species (Baba 2005) demonstrate the outstanding efforts of taxonomists to accurately describe and interpret squat lobster diversity.

The genus *Paramunida* Baba, 1988, recently transferred to the family Munididae (Ahyong et al. 2010), was established by K. Baba to accommodate seven species morphologically close to *Munida* Leach, 1820, but characterized by having a short-rostrum, carapace covered with spinules or granules, indistinct transverse striae, a well-developed distomesial spine on the first article of the antennal peduncle and the male gonopods present only on the second abdominal somite. In following years, several species were described from New Caledonia and adjacent waters (e.g., Cabezas et al. 2009; Macpherson 1993, 1996) and the most recent taxonomic revisions revealed the surprising existence of 14 new species (Cabezas and Chan 2014; Cabezas et al. 2010).

The genus includes 40 genetically distinct yet morphologically very similar species (Cabezas et al. 2010). Interestingly, the species *Paramunida granulata* (Henderson, 1885) is unique in having a granulated carapace and the distomesial spine of antennal article 2 very long, almost reaching the end of the anterior prolongation of article 1 (Baba 1988; Cabezas et al. 2010). A previous phylogenetic study reported this species as the earliest offshoot within the genus in the early Oligocene (Cabezas et al. 2012), which along with the above-mentioned marked morphological differences and a high genetic divergence indicate that this species followed an independent evolutionary trajectory (Cabezas et al. 2010; Cabezas et al. 2012; Machordom and Macpherson 2004). To reflect these findings, we herein propose a new genus, *Hendersonida* gen. n.

Furthermore, during a recent visit to Los Angeles County Museum of Natural History, some *Paramunida* specimens previously identified as *Munida hawaiiensis* (Baba, 1981) were discovered to be an undescribed species. The material examined was collected by the NOAA ship RV *Townsend Cromwell* in Christmas (Kiritimati) Island, Kiribati, in the Central Pacific Ocean. To date, only the endemic species *Paramunida hawaiiensis* (Baba, 1981) from Hawaii, *Paramunida spatula* Macpherson, 2006 from the Austral Archipelago and *Paramunida echinata* Macpherson, 1999 from the Marquesas Islands are known from Central Pacific waters. Therefore, the new species described here is the fourth record of the genus for the region. Finally, we present an updated dichotomous key to species of *Paramunida*.

## Material examined

We studied material collected by the NOAA RV *Townsend Cromwell* Cruise during February–March 1973 in the Central Pacific Ocean. The new described species in this study is deposited in Los Angeles County Museum of Natural History, Los Angeles (LACM). The terminology used mainly follows Baba et al. (2011). The size of the carapace is indicated as the postorbital carapace length measured along the dorsal midline from the posterior margin of the orbit to the posterior margin of the carapace. The length of the antennular and antennal articles is measured excluding distal spines along their lateral margins; the width is measured at midlength of each article. The abbreviations used are: P1 = first pereopod (chelipeds), P2–P4 = second to fourth pereopods (first to third walking legs).

### Molecular data

The phylogenetic tree presented in this study was obtained from Cabezas and Chan (2014). The new species described here failed amplification because material was preserved in formalin, so no molecular comparison is provided.

## Family Munididae Ahyong, Baba, Macpherson & Poore, 2010

### Genus Paramunida

Taxon classificationAnimaliaDecapodaMunididae

Baba, 1988

Paramunida Baba, 1988: 175 (gender: feminine). – Poore 2004: 239. – Baba 2005: 197. – Baba et al. 2008: 171 (compilation of species). – Baba et al. 2009: 277. – Cabezas et al. 2010: 5. – Macpherson and Baba 2011: 60.

#### Diagnosis.

(modified from Baba et al. 2009) Carapace as long as wide; dorsal surface covered with spinules, indistinct transverse striae; posterior margin with some spines; rostrum short, basally subtriangular, distally ending in spine; supraocular spines small, clearly not reaching midlength of rostrum and falling short the end of the corneae; margin between rostral and supraocular spines straight or slightly concave; anterolateral spines well developed at front near anterolateral angles, reaching the level between rostrum and supraocular spines; lateral margins with some spines. Eyes large, maximum corneal diameter about one-third distance between anterolateral spines. Lateral margin of antennular article 1 with distal slender portion about half as long as proximal inflated portion, with 2 distal small spines. Antennal peduncle with anterior prolongation of article 1 spiniform; article 2 with distomesial spine never reaching end of anterior prolongation of article 1. P1–P4 long and slender, squamate; P2–P4 dactyli slender, curved and unarmed on flexor margin. Male gonopods only present on the second abdominal somite.

#### Type species.

*Paramunida setigera* Baba, 1988; by original designation.

#### Remarks.

The *Munida scabra* group was recognized by K. Baba in 1981. It included five species – *Munida scabra* (Henderson, 1885), *Munida granulata* (Henderson, 1885), *Munida proxima* (Henderson, 1885), *Munida tricarinata* (Alcock, 1894) and *Munida hawaiiensis* (Baba, 1981) – all characterized by having a short rostrum, carapace without transverse ridges covered by spinules and granules, the antennal peduncle with a well-developed anterior prolongation of article 1, and male gonopods absent from first abdominal somite. All these peculiarities suggested that the *scabra* group represented an independent lineage from *Munida*, but further investigations were recommended. Later work confirmed the taxonomic significance of this group and the genus *Paramunida* Baba, 1988 was formally described in a report on the chirostylid and galatheid crustaceans from the “Albatross” Philippine Expedition (Baba 1988). This new genus accommodated the species belonging to the *scabra* group plus two new described species *Paramunida longior* and *Paramunida setigera*. *Paramunida* was substantially enlarged through the MUSORSTOM-TDSB expeditions in waters around the Philippines, Indonesia and New Caledonia (Macpherson 1993; Baba 2005), Wallis and Futuna (Macpherson 1996), eastern Australia (Ahyong and Poore 2004), Fiji and Tonga (Macpherson 2004), French Polynesia (Macpherson 2006), New Zealand (Ahyong 2007), Taiwan and Japan (Baba et al. 2009; Macpherson and Baba 2009), and the Solomon Islands (Cabezas et al. 2009). Most recently, the taxonomic revision of the genus resulted in the description of 11 new species (Cabezas et al. 2010), and examination of material collected during the PANGLAO expeditions added three new ones namely *Paramunida akaina*, *Paramunida aspera* and *Paramunida aurora* (Cabezas & Chan, 2014). After the taxonomic rearrangements proposed in the present study the genus *Paramunida* comprises 40 species (see below).

### Paramunida haigae sp. n.

Taxon classificationAnimaliaDecapodaMunididae

http://zoobank.org/5ECE748F-15AA-4AF9-9767-866162CB3B58

Figs 1 , 2 , 3

#### Material examined.

Holotype: Christmas (Kiritimati) Island, Line Islands, Kiribati, 01°51.3'N, 157°30.4'W, February–March 1973, 183 m (NOAA RV *Townsend Cromwell* Cruise): male, 16.6 mm (LACM–CR1973-3312). Paratypes: collected with holotype: 9 males 11.4–17.2 mm (2 broken), 3 females, 13.5–14.1 mm, 2 ovigerous females, 11.6–14.2 mm (LACM–CR1973-3313).

#### Description.

**Figure 1.**
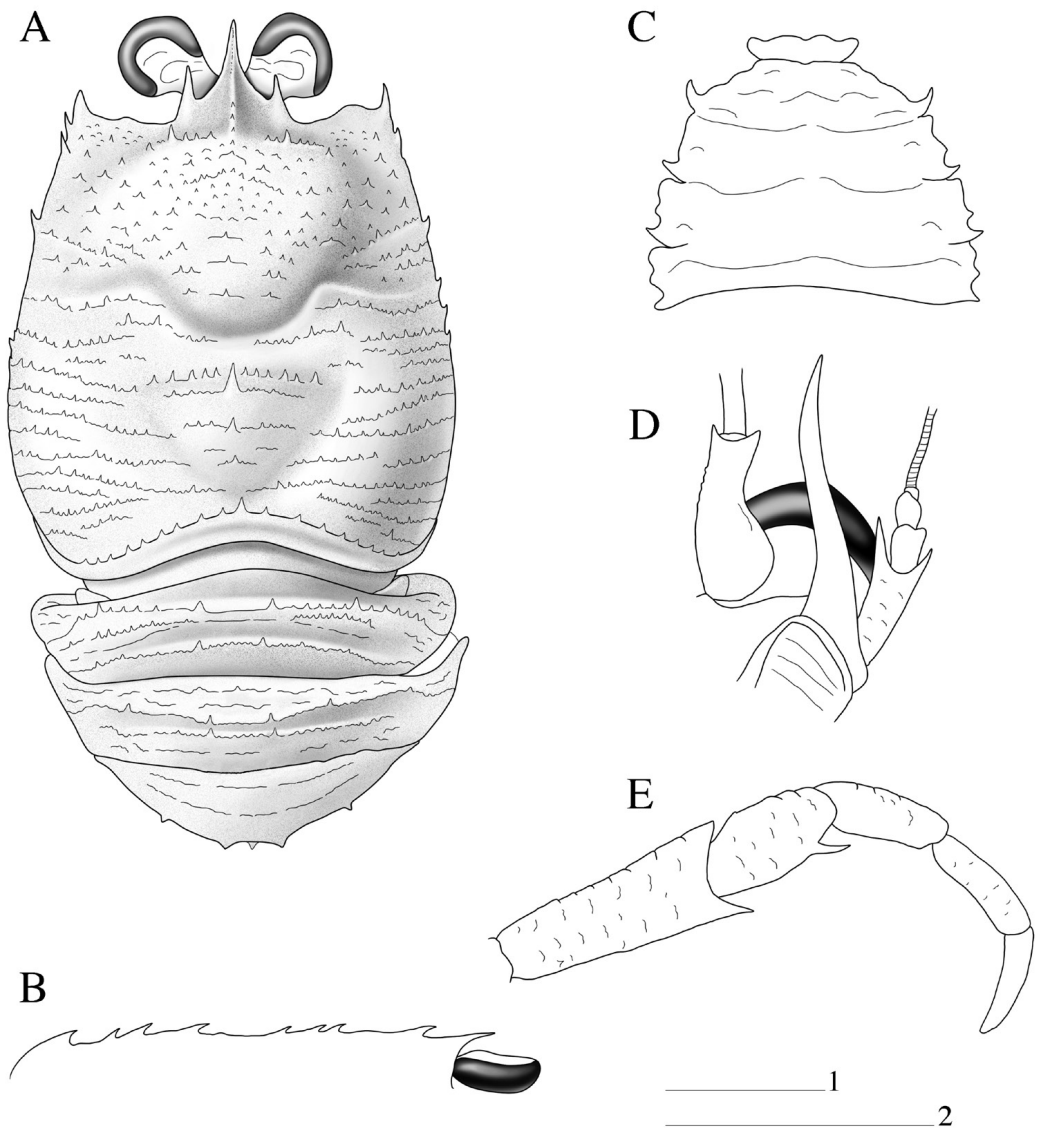
*Paramunida haigae* sp. n. male holotype, 16.6 mm (LACM–CR1973-3312). Christmas (Kiritimati) Island. **A** carapace and abdomen, dorsal view **B** carapace, lateral profile **C** sternum **D** left antennule and antenna, ventral view **E** right maxilliped 3, lateral view. Scale: 5 mm (scale 1 for **A–C, E**; scale 2 for **D**).

Carapace: As long as broad, dorsal surface covered with spinules; each spinule usually on short arcuate striae, with few short uniramous setae. Epigastric region with 2 spines, each behind supraocular spine; with median row of spinules behind rostral spine. Mesogastric region with median row of 3 small spines. Anterior branch of cervical groove with short setae. Cervical groove distinct. Cardiac and anterior branchial regions slightly circumscribed. Cardiac region with a median row of 3 small spines, first thicker than others. Each branchial region with row of spines near cardiac region. Frontal margin slightly concave. Lateral margins convex, with some spines and iridescent setae on anterior half. Anterolateral spine well developed, reaching sinus between rostral and supraocular spines. Rostral spine spiniform, with thin dorsal longitudinal carina; supraocular spines well developed and slender and shorter than rostrum (Figs 1A, B, 3).

Sternum: Thoracic sternite 4 with few arcuate striae; sternites 5–7 smooth (Fig. 1C).

Abdomen: Abdominal somites 2–3 each with 4 well-developed spines on anterior ridge, posterior ridge with 2 median spines. Abdominal somite 4 with 4 spines on anterior ridge; posterior ridge with distinct single median spine. Ridges with numerous spinules and a few small spines (Fig. 1A).

Eyes: Maximum corneal diameter more than one-third distance between bases of anterolateral spines.

Antennule: Article 1 slightly exceeding corneae, with distomesial spine small and as long as distolateral; about twice longer than wide and with fringe of long setae along lateral margin; lateral margin with distal slender portion about half as long as proximal convex portion (Fig. 1D).

Antenna: Anterior prolongation of article 1 overreaching antennular peduncle by about one-third of its length. Article 2 about twice length of article 3 and twice longer than wide, ventral surface with scales; distomesial spine spiniform without tuff of setae, overreaching end of article 3, not reaching end of antennal peduncle, reaching mid-length of anterior prolongation of article 1, and clearly not reaching end of basal article of antennule, distolateral spine not reaching end of article 3; article 3 about 1.5 times longer than wide and unarmed (Fig. 1D).

Maxilliped 3: Ischium about twice length of merus measured along extensor margin, flexor margin bearing long distal spine; merus with well-developed median spine on flexor margin; extensor margin unarmed (Fig. 1E).

**Figure 2.**
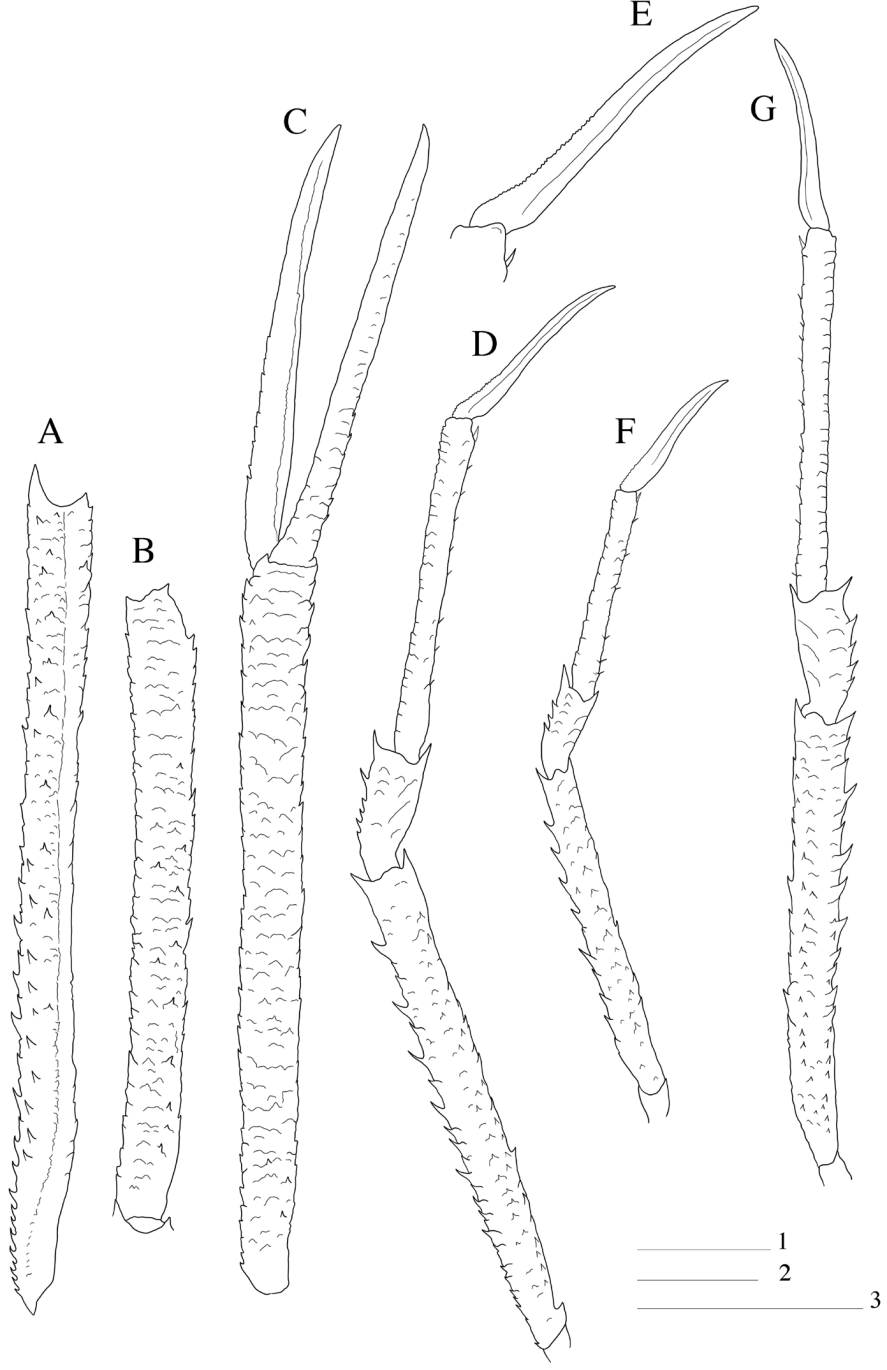
*Paramunida haigae* sp. n. male holotype, 16.6 mm (LACM–CR1973-3312). Christmas (Kiritimati) Island. **A** left merus P1, dorsal view **B** left carpus P1, dorsal view **C** left P1, palm and fingers, dorsal view **D** right P3, lateral view **E** right P3 dactylus **F** male paratype, 11.5 mm (LACM–CR1973-3313), right P2, lateral view. **G** left P4, lateral view. Scale: 5 mm (scale 1 for **A–C, D, G**; scale 2 for **F**; scale 3 for **E**).

Pereopod 1 (cheliped): Long and slender, squamate, between 6.5–7.5 times carapace length; carpus about as long as palm, and 7–10 times longer than high; palm 1.1–1.5 times fingers length. Base of carpus without bundle of setae (Fig. 2A–C).

**Figure 3.**
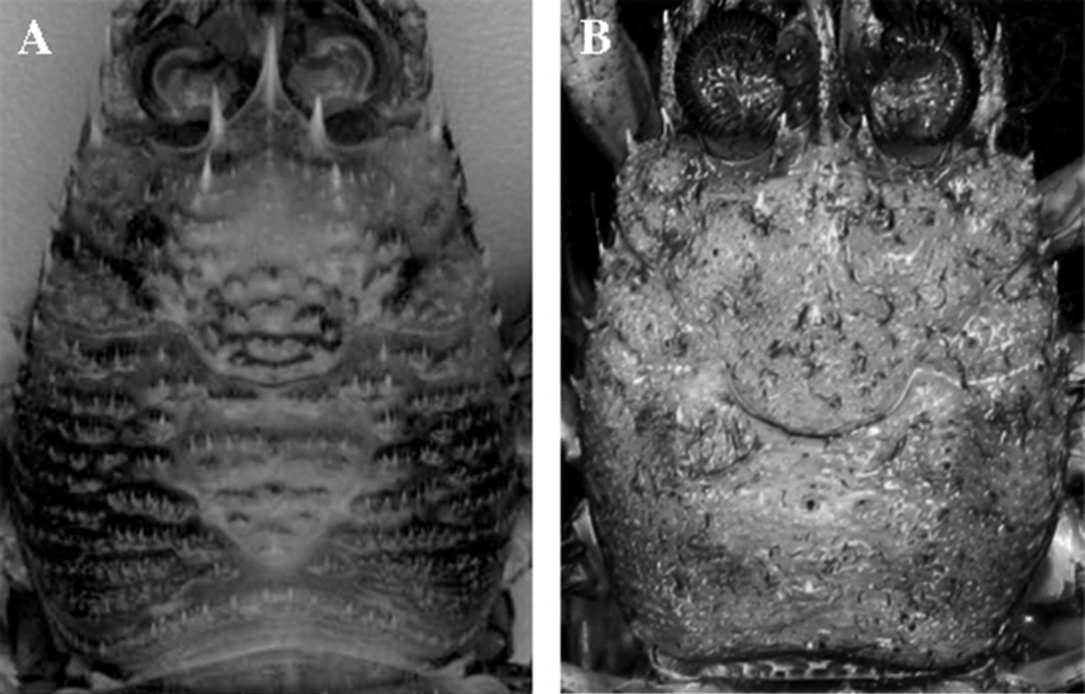
Dorsal surface of the carapace, dorsal view. **A** *Paramunida haigae* sp. n., NOAA *Townsend Cromwell* Cruise, holotype, male 16.6 mm. **B** *Hendersonida granulata*, BATHUS 2, Stn CP735, female, 13.7 mm.

Pereopods 2–4 (P2 lacking in holotype): Long and slender, with scales on lateral sides of meri, carpi and propodi; scales with short setae. P2 2.5–3.5 times carapace length, merus 1.1–1.6 times longer than carapace, about 8–10 times as long as high, 4 times as long as carpus and 1.5 times as long as propodus; propodus about 7–10 times as long as high, and 1.4–1.7 times dactylus length. Merus with well-developed spines on extensor margin, increasing in size distally; flexor margin with few spines and one well developed distal spine; row of small spines along flexolateral margin. Carpus with few small extensor spines, small distal spine on extensor and flexor margin. Propodus with small movable flexor spines. Dactylus compressed, slightly curved, with longitudinal carinae along mesial and lateral sides, flexor border unarmed. End of P2 carpus not reaching end of P1 merus. P3 with similar spination and article proportions as P2; propodus slightly longer than P2 propodus, merus and dactylus as long as those of P2. P4 as long as P2; merus 1.1–1.3 times carapace length; propodus and dactylus slightly longer than those of P3; merocarpal articulation clearly exceeding end of anterior prolongation of article1 of antennal peduncle (Fig. 2D–G).

#### Etymology.

This species is dedicated to the renowned carcinologist Janet Haig (1925–1995) who first classified the material examined.

#### Remarks.

*Paramunida haigae* sp. n. closely resembles *Paramunida antares* Cabezas, Macpherson & Machordom, 2010 from New Caledonia. The new species is readily separated from *Paramunida antares* in having the rostrum spiniform rather than triangular. Moreover, the mesogastric region in *Paramunida antares* has 3 well-developed spines, but these spines are very small in *Paramunida haigae* sp. n. The two species also differ in the article 2 of the antennal peduncle: twice as long as wide in the new species but only 1.5 times in *P. antares.* Finally, the distomesial spine of antennal article 2 clearly overreaches the end of article 3 in the new species, but this spine only reaches the end of the article 3 in *Paramunida antares*.

The new species is also very close to *Paramunida achernar* Cabezas, Macpherson & Machordom, 2010 from Tonga. *Paramunida haigae* sp. n. can be distinguished from *Paramunida achernar* by having 3 small mesogastric spines (vs. 3 well-developed spines in *Paramunida achernar*). Furthermore, the anterior prolongation of antennal article 1 is clearly longer in *Paramunida haigae* sp. n., overreaching the antennular peduncle by about one-third of its length but only by one-fourth in *Paramunida achernar*, and the distomesial spine of antennal article 2 overreaching the end of article 3 in the new species (vs. only reaching the end of the article 3 in *Paramunida achernar*). Finally, the merocarpal articulation of P3 clearly exceeds the anterior prolongation of the antennal article 1 in the new species, only slightly exceeding the anterior prolongation in *Paramunida achernar*.

Of the regional Central Pacific *Paramunida* species, *Paramunida haigae* sp. n. can be easily distinguished from *Paramunida hawaiiensis* Baba, 1981 from Hawaii in having the rostral spine larger than supraocular spines instead of smaller or at most equal to supraocular spines. Furthermore, the distomesial spine of article 2 reaches end of antennal peduncle in *Paramunida hawaiiensis* but never reaches it in the new species. The new species can also be easily distinguished from *Paramunida echinata* Macpherson, 1999 from Marquesas Islands in having the rostral spine spiniform instead of triangular. Finally, *Paramunida haigae* sp. n. is also easily distinguishable from *Paramunida spatula* Macpherson, 2006 from the Austral archipelago by the shape of the anterior prolongation of antennal article 1.

#### Distribution.

Christmas (Kiritimati) Island, Kiribati, at 183 m depth.

### Genus Hendersonida gen. n.

Taxon classificationAnimaliaDecapodaMunididae

http://zoobank.org/C149D702-673C-48D8-BD97-DD6F20A8B59B

#### Type species.

*Munida granulata* Henderson, 1885; here designated and by monotypy.

#### Diagnosis.

Carapace as long as wide; dorsal surface granulose, with some scattered spines and small spinules with short uniramous setae and without transverse ridges; few and short setae along anterior branch of cervical groove; posterior margin with some spines; rostrum spiniform, upturned distally, larger and thicker than supraocular spines; supraocular spines small, clearly not reaching midlength of rostrum and falling short the end of corneae; margin between rostral and supraocular spines straight or slightly concave; anterolateral spines well developed situated at front near anterolateral angles, reaching the level between rostrum and supraocular spines; lateral margins with some spines. Eyes large, maximum corneal diameter about half distance between bases of anterolateral spines. Lateral margin of antennular article 1 with distal slender portion about half as long as proximal inflated portion, with 2 distal spines. Antennal peduncle with anterior prolongation of article 1 spiniform; article 2 with distomesial spine long, almost reaching end of anterior prolongation of article 1. P1–P4 long and slender, squamate; P2–P4 dactyli slender, curved and unarmed along flexor margin. Male gonopods only present on the second abdominal somites.

#### Etymology.

The generic name *Hendersonida* acknowledges the meaningful contributions of John Robertson Henderson (1863–1925) to the field of crustacean taxonomy. Gender: feminine.

#### Remarks.

The carapace dorsal surface devoid of distinct transverse ridges or striae, the rostral spine broad at base, the antennal peduncle with a well-developed anterior prolongation of article 1 and the male gonopods absent from the first abdominal link this new genus to *Paramunida* Baba, 1988. This close relationship has been confirmed by molecular evidence that have rendered this new genus as the sister group of *Paramunida* (Cabezas et al. 2012, Cabezas and Chan 2014). *Hendersonida* gen. n. may be easily differentiated from *Paramunida* by having the dorsal surface of the carapace covered by granules and the distomesial spine of the antennal article 2 almost reaching the end of anterior prolongation of article 1. The genus contains one species.

### Hendersonida granulata

Taxon classificationAnimaliaDecapodaMunididae

(Henderson, 1885)

Fig. 3

Munida granulata Henderson, 1885: 409 (S of the Fiji Islands, 549 m). – Henderson 1888: 133, pl. 14, figs 3, 3a, 3b (off Matuku, Fiji, 576 m).
Paramunida granulata . – Baba, 1988: 176, fig. 72 (Moluccas off W coast of Halmahera, 545 m). – Macpherson 1993: 452, figs 3, 13 (New Caledonia, Loyalty Islands and Indonesia; reexamination of type material; 439–650 m). – Macpherson 1996: 412 (SW Pacific (Futuna Island, Wallis Islands, Bayonnaise Bank), 400–450 m). – Macpherson 2004: 287 (Fiji and Tonga, 395–592 m). – Ahyong and Poore 2004: 68 (Queensland, 548 m). – Baba 2005: 302 (key, synonymies). – Baba et al. 2008: 172 (list of occurrences). – Macpherson and Baba 2011: 60. – Cabezas et al. 2010: 23, fig 13C, 16I (Tonga, Vanuatu, Loyalty Islands, 550–600 m).

#### Diagnosis.

(modified from Cabezas et al. 2010) Rostrum clearly triangular, larger than supraocular spines, with thin dorsal carina; margin between rostral and supraocular spines straight or slightly concave. Minute spinules on gastric and hepatic regions forming groups arising from scale-like striae and with few short uniramous setae. Mesogastric region with 1 well-developed spine. Median cardiac region with 3 or 4 well-developed spines. Few and short setae along anterior branch of cervical groove. Sternal plastron squamate, with numerous striae on sternites 4–7. Lateral margin of antennular article 1 with distal slender portion about half as long as proximal inflated portion. Antennal peduncle with anterior prolongation of article 1 spiniform; article 2 twice longer than broad, with distomesial spine long, almost reaching end of anterior prolongation of article 1, distolateral spine nearly reaching end of article 3; article 3 1.5 times longer than broad. Base of P1 carpus without bundle of setae. P2 propodus 7–8 times as long as wide, and 1.2–1.3 times longer than dactylus.

#### Distribution.

Philippines, Indonesia, Queensland, New Caledonia, Loyalty Islands, Fiji, Tonga, Futuna Island, Vanuatu, Wallis Islands and Bayonnaise Bank, between 395 and 650 m.

#### Remarks.

Detailed illustrations for *Hendersonida granulata* are included in Baba (1988), Macpherson (1993) and the antennule, antenna and dorsal surface of the carapace were newly illustrated in Cabezas et al. (2010).

## Discussion

The present study updates the taxonomy of the genus *Paramunida* Baba, 1988 by describing a new species from the Central Pacific Ocean and transferring one species to a new genus. Deep waters in the Central Pacific Ocean have been poorly sampled and our knowledge on diversity of squat lobster fauna is scarce (Baba 2011; Schnabel et al. 2009). The new species herein described, *Paramunida haigae* sp. n., represents the fourth record of the genus for Central Pacific waters.

**Figure 4.**
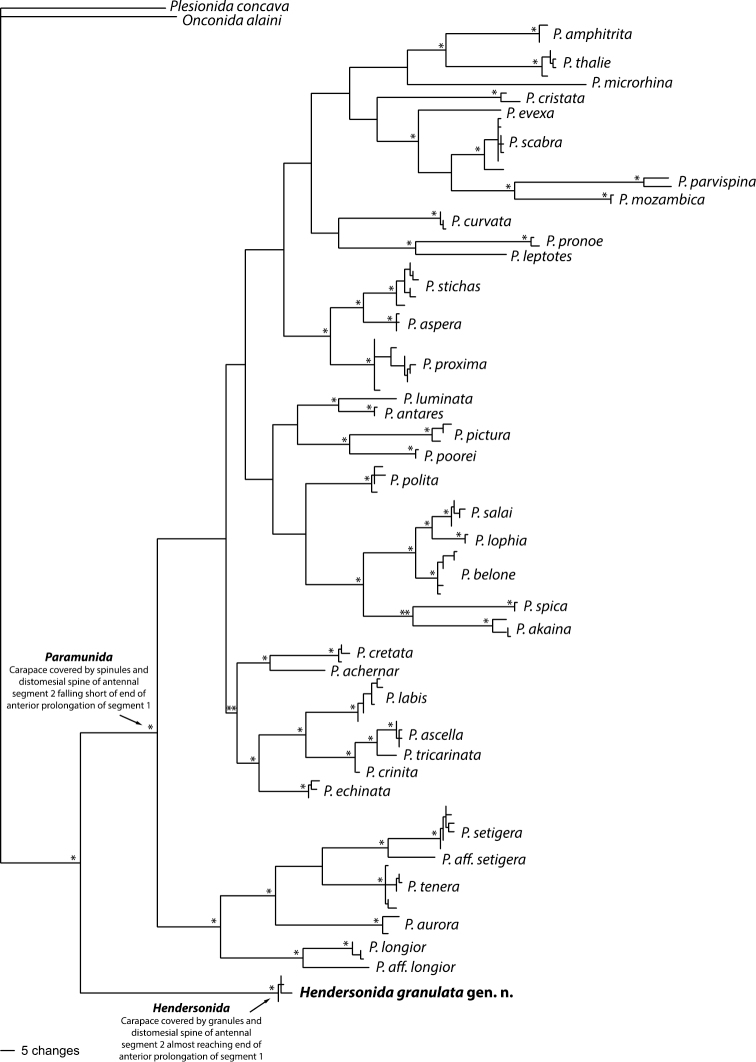
Bayesian tree of the combined dataset (16S + ND1) as modified from Cabezas and Chan (2014). One asterisk represents Pp=1-0.90 and two asterisks Pp=0.70-0.85. *Hendersonida* gen. n. is presented in bold.

The new genus here described contains only *Hendersonida granulata*. Although morphologically very similar to *Paramunida*, recent studies revealed that this species was phylogenetically and genetically very different from the other species of the genus (Cabezas et al. 2010; Cabezas et al. 2012; Cabezas and Chan 2014). This new lineage possesses two conspicuous diagnostic characteristics that make it easy to differentiate from species of *Paramunida*: (1) the armature of the dorsal surface of the carapace, and (2) the length of the distomesial spine of antennal article 2. *Hendersonida* is unique in having a granulated carapace and the distomesial spine of antennal article 2 almost reaching the end of anterior prolongation of article 1 (Cabezas et al. 2010). All other characters present a certain degree of variation among species and they are not useful to distinguish genera. At a molecular level, divergence values between *Paramunida* and *Hendersonida* are within the range cited for other squat lobster genera (Cabezas et al. 2008; Machordom and Macpherson 2004), with a mean divergence of 8.05% for the 16S gene, 18.5% for the ND1 gene and 15.3% for the COI gene. Furthermore, recent phylogenetic studies including mitochondrial and nuclear markers confirmed *Hendersonida granulata* as a highly supported monophyletic clade separated by a long branch from *Paramunida* s.s. and originated at least 10 mya before the radiation of *Paramunida* between 21–17 million years ago (Cabezas and Chan 2014; Cabezas et al. 2012). Based on these findings, our decision to designate a new genus is well supported (Fig. 4).

*Hendersonida granulata* is a widespread species distributed from the Philippines to to Northern Australia and the South Western Pacific, including New Caledonia, Vanuatu, Fiji, Tonga and Wallis and Futuna, between 395 and 650 m. This is unusual, since most deep-sea squat lobsters are characterized by having reduced geographic ranges confined to a single archipelago or a biogeographic area (Schnabel et al. 2011). Previous studies have reported how widely distributed species within deep-sea squat lobsters are most likely to be complexes of species with more restricted distributions (Cabezas et al. 2012; Poore and Andreakis 2011, 2012). For *Hendersonida granulata* only specimens from the South West Pacific (New Caledonia, Vanuatu and Tonga) have been studied in an integrative phylogenetic framework (Cabezas et al. 2012), so future surveys collecting new material through its entire range will help to infer genealogical relationships among geographically distinct populations. The taxonomic rearrangements in this study bring current diversity within *Paramunida* up to 40 species and up to 21 genera within the family Munididae.

### Key to species of *Paramunida*

**Table:** 

1	Anterior prolongation of antennal article 1 spatulate	*Paramunida spatula* Macpherson, 2006
–	Anterior prolongation of antennal article 1 spiniform	2
2	Rostral spine smaller or at most equal to supraocular spines	3
–	Rostral spine larger than supraocular spines	8
3	Margin between rostral and supraocular spines clearly convex	*Paramunida curvata* Macpherson, 2004
–	Margin between rostral and supraocular spines straight or slightly concave	4
4	Antennal article 2 with minute distomesial spine	*Paramunida microrhina* Cabezas, Macpherson & Machordom, 2010
–	Antennal article 2 with well-developed distomesial spine	5
5	Mesogastric region with 3 well-developed spines in midline	*Paramunida hawaiiensis* (Baba, 1981)
–	Mesogastric region with minute spines	6
6	Rostrum triangular	7
–	Rostrum spiniform	*Paramunida aurora* Cabezas & Chan, 2014
7	Sternal plastron with numerous striae. Bundle of setae at base of carpus of P1 present	*Paramunida setigera* Baba, 1988
–	Sternal plastron with few striae on each side of sternites 5–7. Bundle of setae at base of carpus of P1 absent	*Paramunida tenera* Cabezas, Macpherson & Machordom, 2010
8	P2–P4 propodi slender, about 20 times as long as broad	*Paramunida longior* Baba, 1988
–	P2–P4 propodi 7–14 times as long as broad	9
9	Distomesial spine of antennal article 2 mucronated or bluntly produced	10
–	Distomesial spine of antennal article 2 spiniform	23
10	Mesogastric region with 1 (rarely 2) spine	11
–	Mesogastric region with a median row of 3 or 4 distinct spines	14
11	Sternal plastron with numerous striae	*Paramunida proxima* (Henderson, 1885)
–	Sternal plastron with few striae on each side of sternites 5–7	12
12	Distomesial spine of antennal article 2 clearly overreaching antennal peduncle	13
–	Distomesial spine of antennal article 2 nearly reaching end of antennal peduncle	*Paramunida antipodes* Ahyong & Poore, 2004
13	Distolateral spine of antennal article 2 not reaching end of article 3	*Paramunida akaina* Cabezas & Chan, 2014
–	Distolateral spine of antennal article 2 overreaching end of article 3	*Paramunida belone* Macpherson, 1993
14	Distomesial spine of antennal article 2 slightly or clearly overreaching antennal peduncle	15
–	Distomesial spine of antennal article 2 never reaching end of antennal peduncle	20
15	Lateral margin of antennular article 1 with distal slender portion as long as proximal inflated portion	*Paramunida spica* Cabezas, Macpherson & Machordom, 2010
–	Lateral margin of antennular article 1 with distal slender portion about half as long as proximal inflated portion	16
16	Distolateral spine of antennal article 2 exceeding antennal article 3	*Paramunida salai* Cabezas, Macpherson & Machordom, 2009
–	Distolateral spine of antennal article 2 not reaching end of antennal article 3	17
17	Mesial margin of antennal article 2, including distal spine, straight. Rostrum triangular or spiniform	18
–	Mesial margin of antennal article 2, including distal spine, convex. Rostrum spiniform	19
18	Rostrum triangular	*Paramunida ascella* Cabezas, Macpherson & Machordom, 2010
–	Rostrum spiniform	*Paramunida mozambica* Cabezas, Macpherson & Machordom, 2010
19	Distomesial spine of antennal article 2 shorter than rest of article 2. Gastric region with short striae. Antennal article 3 about 1.5 times longer than broad	*Paramunida stichas* Macpherson, 1993
–	Distomesial spine of antennal article 2 as long as rest of article 2. Gastric region with moderate-sized striae. Antennal article 3 about twice longer than broad	*Paramunida lophia* Cabezas, Macpherson & Machordom, 2009
20	Mesogastric region without well-developed spines	*Paramunida parvispina* Cabezas, Macpherson & Machordom, 2010
–	Mesogastric region with a row of 3 or 4 distinct spines	21
21	Sternal plastron with numerous striae. Article 2 of antennal peduncle bluntly produced distomesially	*Paramunida evexa* Macpherson, 1993
–	Sternal plastron with few striae, sternites 5–7 with few striae on each side. Article 2 of antennal peduncle produced distomesially ending in distinct spine	22
22	Rostrum triangular. Propodus of walking legs more than 1.5 times dactylus length	*Paramunida echinata* Macpherson, 1999
–	Rostrum spiniform. Propodus of walking legs slightly longer than dactylus	*Paramunida labis* Macpherson, 1996
23	Rostrum with thick dorsal carina	*Paramunida cristata* Macpherson, 2004
–	Rostrum with thin dorsal carina	24
24	Distomesial spine of antennal article 2 clearly exceeding antennal peduncle	*Paramunida leptotes* Macpherson & Baba, 2009
–	Distomesial spine of antennal article 2 at most reaching end of antennal peduncle	25
25	Mesogastric region with 1 (rarely 2) spine	26
–	Mesogastric region with a row of 3 or 4 distinct spines	29
26	Median cardiac region with 1 spine	*Paramunida pronoe* Macpherson, 1993
–	Median cardiac region with a row of 3 or 4 spines	27
27	Tufts of long and dense setae along anterior branch of cervical groove	*Paramunida crinita* Cabezas, Macpherson & Machordom, 2010
–	Few and short setae along anterior branch of cervical groove	28
28	Sternal plastron with few striae, sternites 5–7 only with few striae on each lateral side	*Paramunida polita* Macpherson, 1993
–	Sternal plastron with numerous striae	*Paramunida scabra* (Henderson, 1885)
29	Sternal plastron with numerous striae	30
–	Sternal plastron with few striae, sternites 5–7 only with few striae on each lateral side	31
30	Antennal article 3 twice as long as broad. Few and short setae along anterior branch of cervical groove	*Paramunida thalie* Macpherson, 1993
–	Antennal article 3 slightly longer than broad. Tufts of long and dense setae along anterior branch of cervical groove	*Paramunida tricarinata* (Alcock, 1894)
31	Distomesial spine of antennal article 2 reaching or slightly exceeding end of antennal peduncle. Distolateral spine of antennal article 2 reaching or slightly exceeding end of antennal article 3	32
–	Distomesial spine of antennal article 2 not reaching end of antennal peduncle. Distolateral spine of antennal article 2 not reaching end of antennal article 3	33
32	Antennal article 3 as long as wide	*Paramunida aspera* Cabezas & Chan, 2014
–	Antennal article 3 about 1.5 times longer than wide	*Paramunida marionis* Cabezas, Macpherson & Machordom, 2010
33	Antennal article 3 more than twice longer than broad	*Paramunida amphitrita* Macpherson, 1996
–	Antennal article 3 as long as broad or at most 1.5 times longer than broad	34
34	Antennal article 2 as long as or more than 3 times longer than broad	35
–	Antennal article 2 at most twice longer than broad	36
35	Distomesial spine of antennal article 2 reaching or slightly overreaching end of antennal article 3. Spinules on gastric and hepatic regions mostly forming groups arising from scale-like striae	*Paramunida pictura* Macpherson, 1993
–	Distomesial spine of antennal article 2 not reaching end of antennal article 3. Spinules on gastric and hepatic regions mostly not in groups, lacking scaly striae	*Paramunida poorei* Cabezas, Macpherson & Machordom, 2010
36	Antennal article 2 slightly longer than broad	*Paramunida cretata* Macpherson, 1996
–	Antennal article 2 twice longer than broad	37
37	Row of small epigastric spines behind rostral spine absent	*Paramunida luminata* Macpherson, 1996
–	Row of small epigastric spines behind rostral spine present	38
38	Rostrum triangular	*Paramunida antares* Cabezas, Macpherson & Machordom, 2010
–	Rostrum spiniform	39
39	Mesogastric region with 3 small spines. Merocarpal articulation of P3 clearly exceeding end of anterior prolongation of antennal article 1	*Paramunida haigae* sp. n.
–	Mesogastric region with 3 well-developed spines. Merocarpal articulation of P3 slightly exceeding end of anterior prolongation of antennal article	*Paramunida achernar* Cabezas, Macpherson & Machordom, 2010

## Supplementary Material

XML Treatment for Paramunida

XML Treatment for Paramunida haigae

XML Treatment for Hendersonida

XML Treatment for Hendersonida granulata
